# Software tools and platforms in Digital Pathology: a review for clinicians and computer scientists

**DOI:** 10.1016/j.jpi.2022.100103

**Published:** 2022-06-03

**Authors:** Rodrigo Escobar Díaz Guerrero, Lina Carvalho, Thomas Bocklitz, Juergen Popp, José Luis Oliveira

**Affiliations:** a BMD Software, PCI - Creative Science Park, 3830-352 Ilhavo, Portugal; b DETI/IEETA, University of Aveiro, 3810-193 Aveiro, Portugal; c Institute of Anatomical and Molecular Pathology, Faculty of Medicine, University of Coimbra, 3004-504 Coimbra, Portugal; d Leibniz Institute of Photonic Technology Jena, Member of Leibniz research alliance ‘Health technologies’, Albert-Einstein-Straße 9, 07745 Jena, Germany; e Institute of Physical Chemistry and Abbe Center of Photonics (IPC), Friedrich-Schiller-University, Jena, Germany

**Keywords:** Digital Pathology, Whole Slide Imaging, Image analysis, Computational pathology, Pathomics

## Abstract

At the end of the twentieth century, a new technology was developed that allowed an entire tissue section to be scanned on an objective slide. Originally called virtual microscopy, this technology is now known as Whole Slide Imaging (WSI). WSI presents new challenges for reading, visualization, storage, and analysis. For this reason, several technologies have been developed to facilitate the handling of these images. In this paper, we analyze the most widely used technologies in the field of digital pathology, ranging from specialized libraries for the reading of these images to complete platforms that allow reading, visualization, and analysis. Our aim is to provide the reader, whether a pathologist or a computational scientist, with the knowledge to choose the technologies to use for new studies, development, or research.

## Introduction

Pathology is a field of medicine that deals with the study and diagnosis of diseases. A disease can be diagnosed by examining organs, tissues, fluids, or in some cases by the examination of the entire body (autopsy). Since the invention of the microscope, pathologists have been able to examine and categorize samples with different degrees of magnification, making it possible to visualize elements imperceptible to the human eye.

One of the most important changes that pathology has undergone in recent decades is the inclusion of digital cameras in microscopes. In the beginning, the digitalization of histological images was only a photograph or video of the microscope field of view. Virtual microscopes, also known as Whole Slide Imaging (WSI), are now used in Digital Pathology (DP).^1^

### Whole Slide Imaging

Whole Slide Imaging (WSI) technology arose from several efforts to digitize whole tissue slides into high-resolution images. The first to describe and carry out this task were Ferreira et al. in 1997^2^, ^3^, ^4^ defining it as a “virtual microscope”. It was not until 1999 that the cost and speed of WSI systems became affordable thanks to the contribution of Wetzel and Gilbertson ^5^^,^^6^ while working for Interscope Technologies, and since then there has been wide interest in its use commercially.

**Fig. 1:**
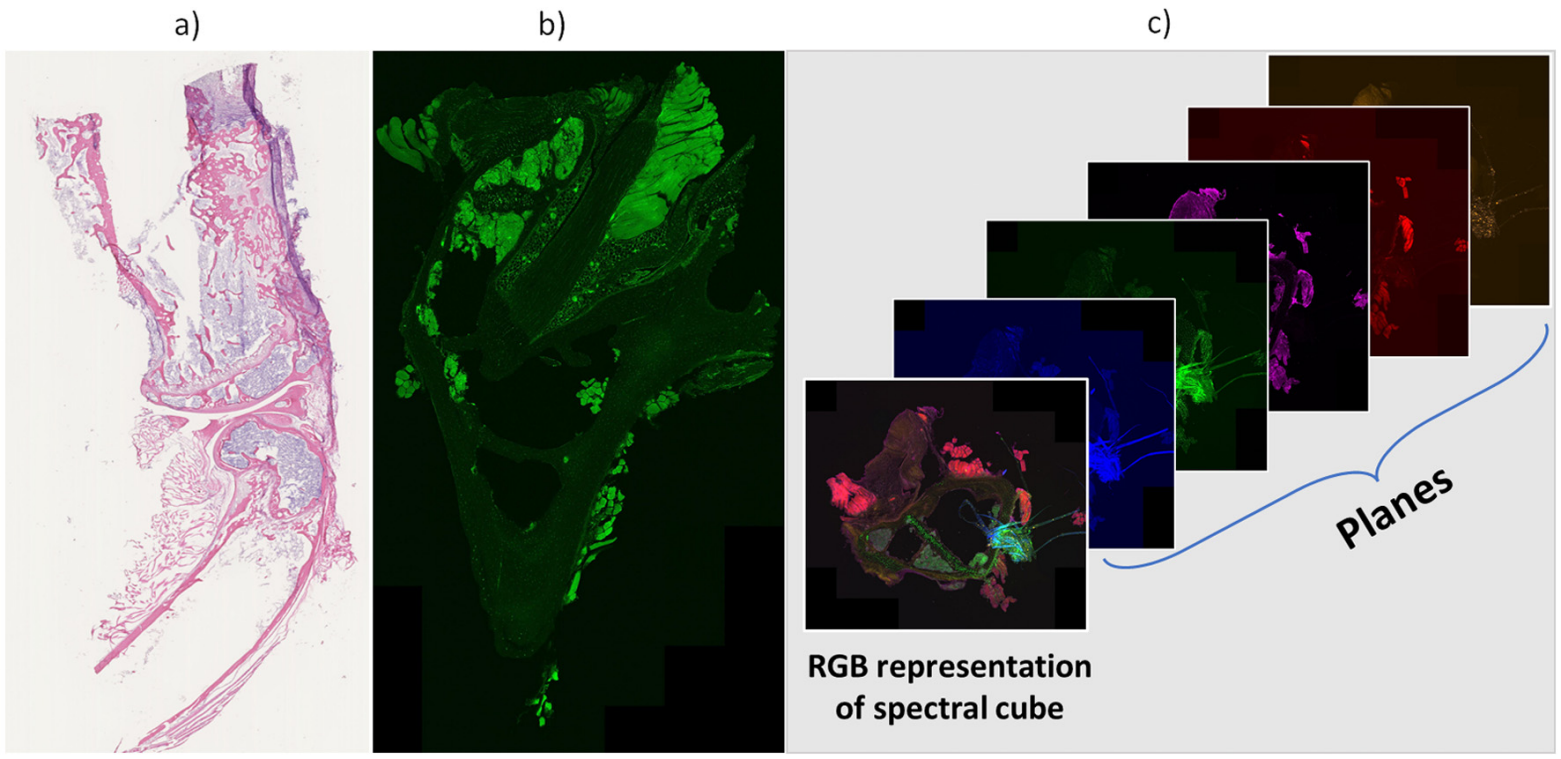
Examples of WSI modalities. (a) Brightfield image, (b) Fluorescence image, and (c) Fluorescence multispectral, planes, and RGB representation cube. These images were provided by the Leibniz-Institutfür Photonische Technologien, and correspond to different hard bone and bone marrow tissues of a mouse.

WSI systems have 4 main elements: (1) a light source, (2) a microscope with multiple lenses, (3) a digital camera, and (4) a system for repositioning the camera view along the sample.^3^^,^^6^^,^^7^ WSI systems capture very high-resolution images (in the range of gigapixels). Those images can be scanned with different magnitudes, most commonly x20 or x40, and higher magnitudes are used in specific cases, e.g. blood smears.^8^ Currently, WSI scanners can use one or several imaging modes, which are bright-field, fluorescent, and multispectral imaging.^6^^,^^8^ Fig. 1 shows different examples of WSI modalities. Each of these imaging modes highlights different anatomical structures or physiological events in the tissue. Different light sources are used in each of these modalities. Bright-field uses a white light within the visible spectrum (Fig. 1a), whereas in fluorescence imaging, tissue is irradiated with light of a certain wavelength, which then emits lower-energy light^9^ (Fig. 1b). Spectroscopic images are obtained by acquiring the spectral information of each pixel in an image. This information is called a data cube because it not only contains information on the x and y axes but also each z plane contains intensity information at various wavelengths, creating a stack of spectral images. An example of an RGB representation of the spectral cube with its component planes can be seen in Fig. 1c.

**Fig. 2:**
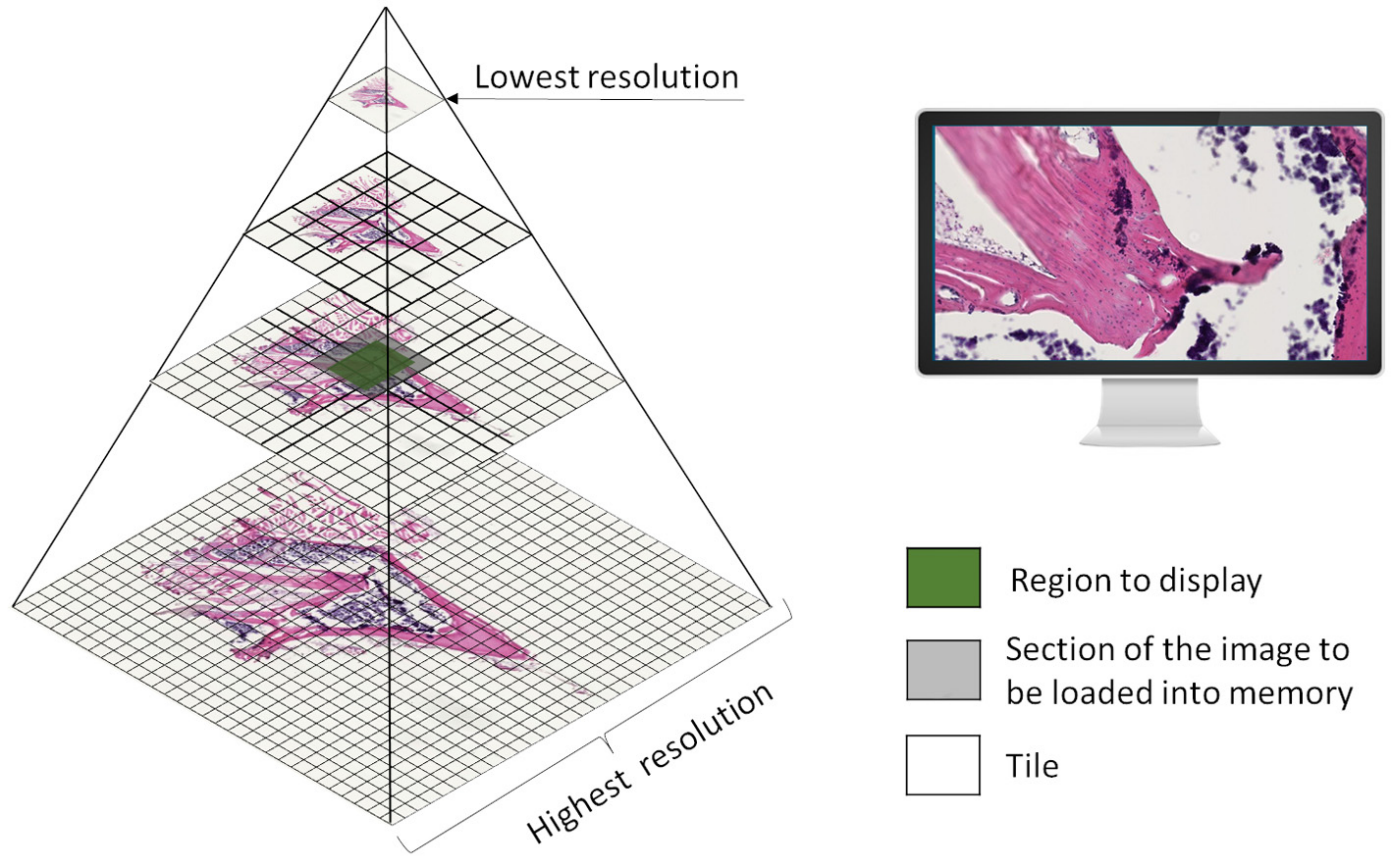
Pyramid organization used to store and visualize WSIs. This illustration shows a pyramid representation of a whole slide image with 4 levels. The top-level of the pyramid has the lowest resolution, the bottom level has the highest resolution, and the middle levels have intermediate resolutions. Each level is divided into two-dimensional blocks called tiles (unfilled squares). This image also shows the tiles that will be loaded in memory (Gray squares) to display a region of interest (Green squares). The resolution used (pyramid level) will depend on the zoom that is requested.

The most common way to display a two-dimensional image on a computer is by a single frame organization, where all the pixels of an image are stored in rows. This organization has limitations when very high-resolution images are involved, so a multi-resolution pyramid method of organization is used instead. To create a pyramid image, it is necessary to replicate the image in multiple resolutions that are called pyramid levels, then each of these levels is divided into two-dimensional blocks of pixels of the same size called tiles. Only the tiles of a pyramid level involved in the viewing area will be loaded into memory. Fig. 2 shows the different elements of a pyramid organization and the tiles that will be loaded into memory when a specific area of the image is visualized. Not all software that we explore in this paper has support for pyramid organization as we see in the coming sections.

### Image formats

Currently, there is no data format for WSIs that is widely accepted by WSI scanners manufacturers, which led to the creation of a several proprietary file formats, e.g., NDPI, SVS and SCN.^10^ However, 2 candidate formats intend to become the standard for WSIs: (1) Digital Imaging and Communications in Medicine (DICOM) and the (2) Tagged Image File Format TIFF.^11^ In 2010, the DICOM Standard Committee Working Group 26 issued a press release (supplement 145) describing a standardization of Whole Slide Microscopic Image.^12^^,^^13^ This standard includes image storage, image compression, reference of the coordinate system, Z-plan management, workflow management, among others. Therefore, many companies have started to include it in their new systems.The alternative of use DICOM is the TIFF format, in particular the variant created by the Open Microscopy Environment (OME) Consortium in 2005, that allows the use of metadata and simplified the use of single and multiple pyramid images.

As many authors,^3^^,^^8^^,^^11^^,^^14^^,^^15^ we believe that DICOM will become the dominant format, since DICOM not only defines an image format but also defines protocols for image exchange and maintenance that have been used for years in radiology. It is important to remark that DICOM has a mechanism to share pixel data content with other file formats such as TIFF. This feature, called Dual-Personality, allows files to be read as DICOM or as a different format, which provides high compatibility with systems that do not read DICOM files.^16^

Acceptance of the DICOM standard will be gradual as upgrading one of these systems is a very expensive investment, making the conversion from proprietary formats to DICOM a better option. For this task, some platforms use Orthanc software, a cross-platform tool that allows the conversion of WSIs to DICOM following supplement 145. Not all platforms for viewing and analysis of digital pathological images perform this conversion. Instead, they use libraries that facilitate the reading and writing of multiple formats. The most common libraries to perform this task are Bio-Formats and OpenSlide. Bio-Formats is a library developed in java by the Open Microscopy Environment consortium.^17^^,^^18^ Currently, supporting the reading of 158 formats (most of them are referred to other types of biological images). OpenSlide, a library developed in C by Carnegie Mellon University,^19^ was specifically designed to read WSIs. It can currently read 14 different virtual slide formats. Some of the scanner's formats are only readable by one of the two libraries, so it is not uncommon to use both libraries together.

In addition to the different formats that each library can read, there are also differences in how they work. Although it tends to read files faster, OpenSlide is limited to 8-bit, RGB images and only 2 dimensions. Bio-Formats reads files slower, but allows the reading of multidimensional images and is less user-friendly in its implementation.

### Image analysis

**Fig. 3:**
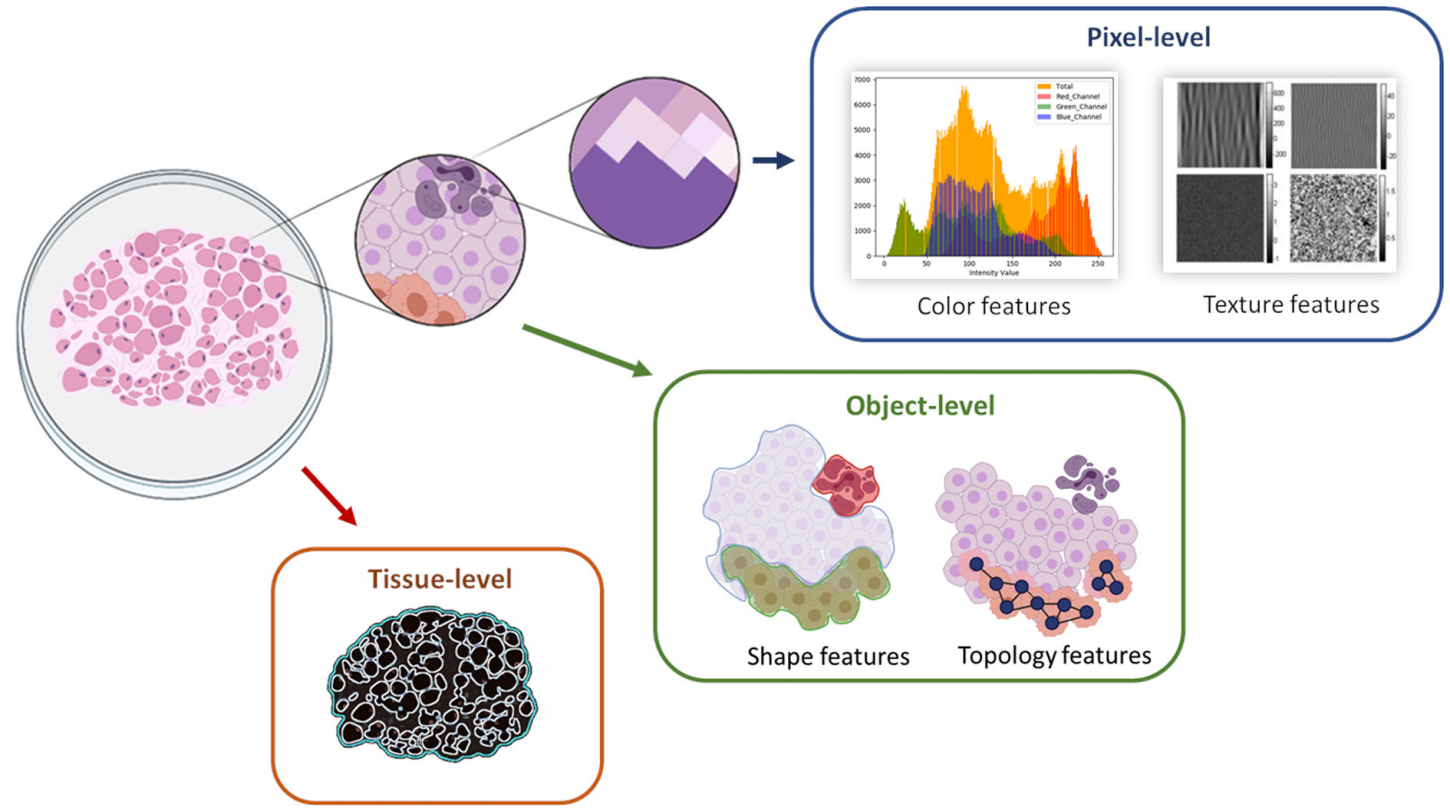
Categorizing of image analysis levels according to magnification. The identification and extraction of the features in the tissue are carried out at different levels of magnification (pixel, object, and tissue levels). At the pixel-level, it is possible to analyze changes in intensity, edge discontinuities, frequency in the histogram, or saturation values. At object-level, objects can be categorized by their morphological characteristics or by their topology. And at the tissue-level, the entire tissue structure is analyzed.

Since WSIs provides large amount of data that can be extracted and analyzed, it is common to perform the analysis focused on cellular information or on region information. When studying cells, we can classify parts of their components such as the nuclei or cytoplasm, classify them by their type (Granulocytes, monocytes, fibroblast, etc.), or analyze the topology to find areas with a higher density of a particular type of cell, namely stroma lymphocytes or structures such as vessels and respective characteristics. We can also look into regions to identify abnormalities in the tissue, such as necrosis or a pre-neoplastic epithelium. The extraction of features from the tissue can be carried out at different levels of magnification, e.g., at the pixel level, at the object level, or at the tissue level (Fig. 3). Frequently, the information from lower levels is used for the analysis of higher levels. At the pixel-level, it is possible to analyze changes in intensity, edge discontinuities, frequency in the histogram, or saturation values. At the next level, objects, such as cells, can be categorized by their morphological characteristics or by their topology. Finally, at the tissue-level, the entire tissue structure is analyzed and large sections of tissue are usually classified.

### Challenges in the image analysis

**Fig. 4:**
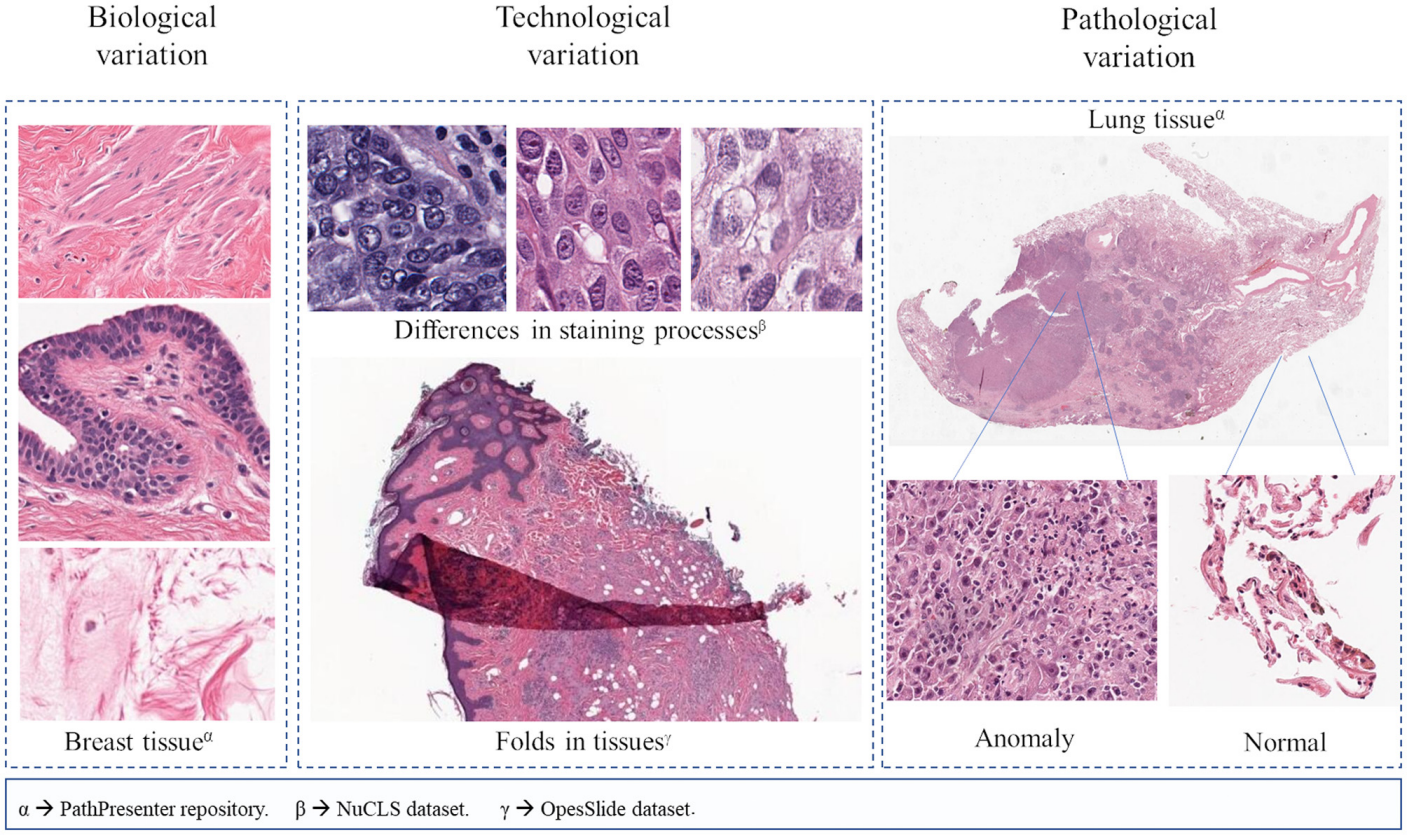
Examples of different types of variation in tissue images. On the left of the image, different sections of the same tissue sample are shown, and one can see variations in the structure and the elements of the same sample. In the middle box, there are shown variations caused by different staining techniques using the same type of tissue (breast). Moreover, a fold in the tissue which makes distortions in shape and color is shown. On the right side, a tissue with healthy areas and areas with pathological alterations is shown.

The image analysis in DP is not a simple task and presents multiple challenges. Firstly, there is great sample variation, which can be classified into 3 categories (Fig. 4): (1) biological variation, (2) pathological variation, and (3) technological variation.^20^ Biological variation refers to the different types of cells that may be present in a sample, in addition to a large number of cellular an tissue elements with varying appearances. Pathological variation refers to all the possible characteristics that tissue may present after inflammation, infection, or alterations that are included in carcinogenesis. Finally, technological variation is caused by the lack of a standard in image acquisition, the technician’s experience, and differences in staining processes.

A second challenge is the size of DP images. The size of whole slide images (WSI) depends on their magnification (zoom level) and sensor pixel size (micron per pixel), but is always in the order of gigapixels.^21^ These sizes complicate the storage, transfer, and analysis of WSIs. As we will see in next sections of this paper, many modern tools only analyze small regions of interest instead of the whole slide.

Over the years, different algorithms have been created to face the actual challenges. Currently, the use of Machine Learning has increased, as this has generated good results in the analysis, segmentation, and classification of microscopic tissue features,^22^, ^23^, ^24^, ^25^, ^26^ mainly when supervised deep learning algorithms are used.^27^, ^28^, ^29^ The supervised algorithms require a large amount of data and their respective annotations, also called ground truth. In the case of pathological images, the annotations cannot be made by people who are not experts in the disease area, which makes it very difficult to obtain these annotations, and in many cases, the datasets cannot be published because they contain sensitive clinical information about a patient.^20^ In addition, there is a great diversity of elements that can be labeled, so that the available databases usually have labels with only one characteristic of the image, e.g., lymphocytes, tumor, fibroblasts, etc. For these reasons, some of the tools that will be described in this paper for the analysis of WSIs do not still present supervised algorithms.

## Software platforms specifically designed for Digital Pathology

The great challenges regarding the visualization, storage, and analysis of WSIs have been presented, and in this section, we will discuss platforms specifically designed to overcome these challenges. These platforms can be divided into 2 main groups: (1) closed-source platforms and (2) open-source platforms. The closed-source platforms are further divided into platforms created by the scanner manufacturers and platforms external to the scanner manufacturers. It is very common for scanner manufacturers to develop their tools for the visualization, manipulation, and analysis of WSIs. Examples of such software are AperioImageScope (Leica Biosystems), HCImage Hamamatsu software (Hamamatsu Photonics), and OptraASSAYS (OptraScan). On the other hand, there are closed-source platforms developed by third parties, e.g., Image-Pro (Media Cybernetics), APP Center (Visio Pharm), Aiforia, Pathobox, ^30^ and Halo image analysis platform (Indicalab). Since all of these are closed architectures, it is impossible to add functions as an external user, and most of them are too expensive for researchers, students, or small laboratories. Open-source platforms give us access to the source code and allow us to add new functions to suit our needs.

Open-source projects involve different members who may participate actively as contributors or passively as end users providing feedback to the contributors. These members are called community members. Large communities allow for more consistent software updates, as well as better support for bug fixes. In DP the open-source platforms with the most developer support are Digital Slide Archive (DSA),^31^ Quantitative Pathology & Bioimage Analysis (QuPath), ^32^ Orbit Image Analysis,^33^ and Cytomine.^34^

DSA is a web-based platform that allows the storage, management, and visualization of WSIs, as well as integrating tools for image annotation and analysis. It was created at the University of Atlanta and is based on CDSA (Cancer Digital Slide Archive).^35^ DSA uses several technologies for its operation; Girder as a base system (open-source, web-based, developed by Kitware), MongoDB as a database system, and Memcached, a distributed memory object caching system that allows fast access to tiles. A proprietary worker management system is used to manage requests for image processing and analysis, while RabbitMQ is used for the workers’ communication. Finally, it integrates HistomicksTK, a toolkit developed in Python for the processing and analysis of WSIs. This contains a rich set of algorithms for pre-processing, segmentation, feature extraction, and many other utilities. One of the major advantages of DSA is that it allows working collaboratively with multiple users in different geographical locations. To handle very high-resolution images, a plugin for girder was created, based on OpenSlide. DSA's ease of use and excellent annotation system has led many researchers to choose it as their primary annotation system^36^^,^^37^ or to create new datasets.^38^

QuPath is a desktop cross-platform tool written in Java and created by Queen's University Belfast. It allows reading of a wide range of formats as it incorporates Bio-Formats, OpenSlide, and DICOM, following supplement 145 (with a pyramid organization). Besides being widely used for DP applications, QuPath is also used in oncology, cell biology, and other areas related to bio-imaging. QuPath enables quantification of various biomarkers, tools for annotation and visualization of WSIs, batch processing, and object and pixel classification using machine learning techniques.^39^ It also incorporates a workflow in bright-field and fluorescent images. In addition, QuPath allows file exchange with ImageJ and MATLAB.

Orbit Image Analysis is a Java software created by Actelion Pharmaceuticals Ltd, now Idorsia Pharmaceuticals Ltd. To read WSIs, it uses Bio-Formats and it supports bright-field and fluorescence multi-channel images. Orbit's analysis focuses on pixel classification, object segmentation, and object classification. The algorithms implemented for the analysis use different machine learning techniques such as Support Vector Machine (SVM) or deep learning. Other algorithms can be easily added even if they were not designed for processing WSIs, thanks to its Map-reduce paradigm, which allows it to process images at the tile level. There are three ways to add new algorithms: by using plugin extensions, the Groovy script editor built into the interface, or an external application such as CellProfiler or ImageJ. Orbit can work with files locally or remotely using the image server OMERO,^40^ which means it can work in collaborative environments.

Cytomine is a web platform originally developed at the University of Liège. Cytomine is very complex software that uses many technologies, but its deployment is automated through the use of Docker containers (lightweight and modular virtual machines), which makes it easy to deploy. The architecture of Cytomine is divided into four main groups; The core server, which as the name indicates is the core of the system containing the technologies concerning the backend of the web application.The Image Management System is a group of image servers and libraries used in the system, e.g., OpenSlide for reading different WSI formats. The user interface is a combination of several technologies that create the user interface and allow communication with other modules via HTTP requests.The data mining module includes different clients (Python and Java) that contain base functions to access resources externally within Cytomine. This module is used to add new analysis algorithms. Cytomine allows simultaneous remote connections to a wide range of collaborative activities and also supports multiple image modalities including multispectral data.^41^

Currently, there are many other open-source applications designed specifically to work with WSIs, but their community is not as wide as the previous ones, so they are limited in their development. However, here we mention some of them, as they may be used more in the future: Automated Slide Analysis Platform (ASAP), Pathology Image Informatics Platform (PIIP),^42^ caMicroscope, slideToolkit,^43^ and Decision Support Tools (desuto).^44^

## General bio-imaging software used in Digital Pathology

In addition to tools specifically developed for the analysis of DP images, bio-imaging platforms are widely used in the field of DP. These platforms often have limited tools for reading, visualizing, and analyzing WSIs. This paper explains in detail the three most widely used: ImageJ, CellProfiller, ICY, and Ilastik; but other tools with similar properties are also mentioned.

ImageJ is a public domain program for image processing. The first version of ImageJ was released in September 1997, and since then it has become one of the most widely used programs in the biological field.^45^ The structure of ImageJ allows the addition of extra modules (plugins and macros) that extend its functionalities. Currently, there are so many modules that some distributions group the most relevant ones in a single package, the most used of these distributions being Fiji ^46^ as it has hundreds of plugins. In 2009, the ImageJ2 project was created with the purpose of improving the core of the first version. This second version has backward compatibility with previous modules thanks to the implementation of the Legacy bridge.^47^ ImageJ allows the integration of Bio-Formats so that the reading of WSIs is possible (limited to the support of Bio-Formats). Unlike specialized programs for handling WSIs, imageJ loads WSIs in memory as if they were images with megapixel resolutions, which results in poor performance when viewing images with higher resolutions. However, there is a plugin called SlideJ,^48^ which improves the workflow when working with WSIs and allows processing at multiple magnifications.

CellProfiller is cell image analysis software started by Anne E. Carpenter and Thouis R. Jones in the Sabatini Laboratory and Golland laboratory.^49^ It was released in 2005 and has since undergone a series of improvements. It was originally developed in MATLAB but in the second version released in 2011 it was rewritten in Python. Its third version enabled support for three-dimensional analysis and deep learning.^50^ The fourth version improved its performance by migrating from Python 2 to Python 3, as well as improving several methods for image analysis. Over the years, CellProfiler has become one of the most widely used forms of software for microscopy image analysis, thanks to its versatility, continuous improvements, and ease of use. One of the main features of Cellprofiller is that it works with individual module pipelines. Each module performs a process on the image (e.g., a crop, identification of a particular object, a color operation, etc.) and is executed sequentially; and these modules can be modified and adapted to the user's needs in a very simple way. However, it does not natively have methods for working with WSIs, so external software (e.g., orbit) is often used to split WSIs into tiles that are then sent to CellProfiler for analysis.

ICY is an open-source software created in 2011 by the BioImage Analysis Lab at Institut Pasteur. It was developed with the goal of covering the widest variety of biological applications, providing easy access to state-of-the-art algorithms for image analysis, and encouraging reproducibility research.^51^ ICY is desktop software written in Java but is complemented by a website that provides a centralized repository for contributing and sharing plugins, scripts, and protocols. The user interface contains a ribbon-style toolbar very similar to the one used in Microsoft's Office Suite, making it very easy to use. ICY integrates Bio-Formats for reading images so it can load WSIs, but it does not allow multi-resolution pyramid organization, so the reading of very high-resolution images has a memory management problem. However, there is a plugin that allows the union of ICY with cytomine called Icytomine.^52^ This plugin establishes a bridge between both applications in order to apply the analysis tools of ICY in WSIs.

Ilastik is a tool specialized in the segmentation, classification, tracking, and counting of elements within biological images.^53^ It has multiple machine learning tools for image analysis. Its operation is similar to that of CellProfiler as it uses pipelines for its workflow and, like CellProfiler, it has a batch processing system where multiple images can be analyzed following this pipeline. In some tasks like object classification, tracking, or pixel classification, Ilastik accepts as input images up to 5D. Although it cannot read WSIs, it can be used to send a batch of the tiles that make up the image for further analysis.

In addition to the tools mentioned above, other authors^15^^,^^44^^,^^51^ mention other tools for the analysis of WSIs. Although these tools or platforms can be easily found and have immense potential, they have not been updated in years. Some of these tools are; BioImageXD^54^ and Bisque.^55^

As an additional suggestion, we would like to recommend subscribing to Image.sc, a scientific discussion forum that gathers tools for Bio-image Analysis. In this forum, it is very common for the creators of the most popular open-source tools and other members of the community to help in solving technical problems.

## Discussion

In recent years, the Digital Pathology Association has created several publications called concept papers. These publications are usually reviews on introductory topics to the world of digital pathology. In Aeffner et al.,^56^ it is mentioned that there are several computational tools for histopathology image analysis, that there are both open-source and commercial tools, and in which cases it would be convenient to use any of these types of tools. However, the tools are never mentioned or analyzed, unlike the present paper.

In addition to the publications of the Digital Pathology Association, other authors have mentioned the different open-source tools used in DP, such as Marée,^57^ which refers to several open practices and resources that can be applied in DP. However, the review of the tools is very superficial and biased towards collaborative elements. Some other reviews have addressed applications for a specific task, e.g., in Korzynska et al.^58^ tools used for cell annotation are analyzed or in Lucas et al.^59^ different deep-learning software used for segmentation is analyzed.

This paper aims to provide an actual compilation of computational tools that both pathologists and computer scientists can use, highlighting their main features and weaknesses. In addition, it describes the different challenges that can be encountered in DP.

Reading WSIs is one of those challenges as there is a wide variety of formats. Libraries such as OpenSlide or Bio-Formats allow reading a large number of them, but none of the libraries still provides support for all existing formats in the market. From our point of view, the best option is the combination of them to cover as many formats as possible.

The next challenge is visualization. Due to the very high resolution of WSIs, a pyramidal organization method is needed. Some general bio-imaging tools like ICY integrate libraries for reading WSIs, but not a pyramidal organization method for visualization, so when trying to display large images there will be problems in memory management. Some other general bio-imaging tools use additional plugins or external software to improve performance. However, the use of platforms specifically designed for DP provides a better experience in viewing WSIs than general bio-imaging tools.

In addition to image reading and visualization, most of the platforms specifically designed for DP provide biomarker annotation and extraction tools, but each one uses different approaches and methodologies that provide distinct differentiating elements. Platforms such as Cytomine or DSA facilitate remote collaboration so that experts located in different parts of the world can work at the same time with the same slide. Qupath, unlike other platforms, has a large number of tools for tissue microarray analysis. Orbit was designed for sophisticated image analysis and integrates several modules to facilitate this task. Table 1 compiles the main characteristics of each of these platforms. In the contributors section, we decided to consider only the contributors of the main repository, but in many of these platforms, there is more than one repository. In the last column, we created a popularity ranking with 1 being the most popular and 4 the least popular. As a metric, we used the number of citations with their reference papers in Google Scholar.

**Table 1:** WSIs platforms comparison

Platform	Type of platform	Main programming language	WSIs reading system	Integrates collaboration tools	Data storage technologies	Default type of analysis	Imaging modalities	License	Contributors in the main repository	Releases year	Popularity ranking
DSA	Web-base	Python	OpenSlide	Yes	Mongodb	Pre-processing, segmentation and feature extraction	Bright-field	Apache-2.0 License	11	2020	3
QuPath	Desktop	Java	Bio-Formats and OpenSlide	Partial	OMERO	Object and pixel classification	Bright-field and fluorescent images	GPLv3 license	9	2016	1
Orbit	Desktop with some web properties	Java	Bio-Formats	No	SQLite and OMERO	Pixel classification, object segmentation and object classification	Bright-field and fluorescence multi-channel images	GPLv3 license	4	2016	4
Cytomine	Web-base	Goovy/Java (Cytomine-Core)	Bio-Formats and OpenSlide	Yes	Mongodb and PostgresSQL	Image classification, semantic segmentation, and landmark detection	Bright-field, fluorescent images and multispectral data	Apache-2.0 License	10	2016	2

Although the platforms specifically designed for DP provide multiple tools for image analysis, in many cases, this analysis does not fit users requirements, so different external general-purpose software is often integrated. This is the case of Icytomine, which combines the power of the algorithms developed in ICY with the collaborative capacity of Cytomine, or the frequent integration of ImageJ with other tools for image processing. Table 2 shows the 4 main general Bio-Imaging software used for image analysis in WSIs, their main characteristics and how they are adapted to work with WSIs.

**Table 2:** Bio-imaging software used in DP comparison.

Tool	Way to work with WSIs	Main programming language	Machine learning tools	Main feature
ImageJ	Using SlideJ	Java	With plugins	It has a wide range of tools for image processing and has the largest community of users and developers
CellProfiller	External software	Python	Yes	Versatility, continuous improvements and ease of use by using pipelines process
ICY	Using the plugin Icytomine or an external software	Java	With plugins	Cover a wide variety of biological applications and have cut edge algorithms
Ilastik	Using external software	Python	Yes	The segmentation, classification, tracking and counting of elements are its speciality

The type of algorithms used for the analysis of DP images is different in each of the computational tools, but the vast majority of them use machine learning algorithms, which have shown excellent results, particularly deep learning models. However, for most deep learning models, a large amount of correctly labeled data is required for training. Unfortunately, in DP, these annotations can only be done by experts, which makes it very difficult to obtain extensive databases. In addition, sensitive patient information is often included, meaning they cannot be publicly accessible. All these problems create a lack of good quality annotated datasets that could improve the performance of supervised algorithms.

There is clearly no perfect platform supporting all formats, all modalities, using the best analysis algorithms, and allowing extensive collaboration with other users. However, we can choose the best platform or tool for our needs and complement it with the functionalities required.

Finally, we would like to mention that the integration of computational tools in the area of pathology aims to help pathologists to visualize and analyze the different characteristics of a tissue as well as enhance their work. The role of pathologists will always be indispensable in improving these tools and under no circumstances is it replaced by any of them.

## Conclusions

This work was focused having in mind 2 targeted users: (1) computational scientists who enter the passionate world of digital pathology for the first time, and (2) pathologists who seek different tools to enhance daily diagnostic, therapeutic scores, and research tools. For the first type of reader, we have described how Whole Slide Images (WSIs) are visualized, which open-source tools can be used to read or convert the different kinds of existing formats, which tools of analysis exist, and the challenges presented by the analysis of WSIs, especially when machine learning algorithms are used. For the second group of readers, we presented a wide range of tools that can be used in their routine research, which tools can segment an image or classify objects, which tool provides a collaborative environment, or simply which tool allows visualization of WSIs in a multi-resolution pyramid organization.

From this analysis, we conclude that the combination of multiple technologies might be the best option to afford the big challenges in Digital Pathology. We believe that in the future, these tools should focus on improving 5 main areas: (1) the use of the DICOM standard, (2) the versatility in the management of multiple imaging modalities (bright-field, fluorescent, and multispectral imaging), (3) the inclusion in the base software of robust algorithms for tissue analysis and diagnosis, (4) the improvement of collaborative tools, and (5) the creation of more user-friendly interfaces for end-users. To improve these areas, pathologists must be actively integrated into development communities providing valuable feedback.
